# Draft genome sequence of *Streptomyces* sp. TP-A0867, an alchivemycin producer

**DOI:** 10.1186/s40793-016-0207-1

**Published:** 2016-10-24

**Authors:** Hisayuki Komaki, Natsuko Ichikawa, Akio Oguchi, Moriyuki Hamada, Enjuro Harunari, Shinya Kodani, Nobuyuki Fujita, Yasuhiro Igarashi

**Affiliations:** 1 Biological Resource Center, National Institute of Technology and Evaluation (NBRC), Chiba, Japan; 2 NBRC, Tokyo, Japan; 3 Biotechnology Research Center and Department of Biotechnology, Toyama Prefectural University, Toyama, Japan; 4 College of Agriculture, Academic Institute, Shizuoka University, Shizuoka, Japan

**Keywords:** Alchivemycin, Biosynthetic gene cluster, Genome mining, Polyketide synthase, *Streptomyces*, Taxonomy

## Abstract

*Streptomyces* sp. TP-A0867 (=NBRC 109436) produces structurally complex polyketides designated alchivemycins A and B. Here, we report the draft genome sequence of this strain together with features of the organism and assembly, annotation, and analysis of the genome sequence. The 9.9 Mb genome of *Streptomyces* sp. TP-A0867 encodes 8,385 putative ORFs, of which 7,232 were assigned with COG categories. We successfully identified a hybrid polyketide synthase (PKS)/ nonribosomal peptide synthetase (NRPS) gene cluster that could be responsible for alchivemycin biosynthesis, and propose the biosynthetic pathway. The alchivemycin biosynthetic gene cluster is also present in *Streptomyces rapamycinicus* NRRL 5491^T^, *Streptomyces hygroscopicus subsp. hygroscopicus* NBRC 16556, and *Streptomyces ascomycinicus* NBRC 13981^T^, which are taxonomically highly close to strain TP-A0867. This study shows a representative example that distribution of secondary metabolite genes is correlated with evolution within the genus *Streptomyces*.

## Introduction

*Actinomycetes* are known for their ability of producing a variety of secondary metabolites with useful pharmacological potency such as antimicrobial, antitumor, and immunosuppressive activities. In particular, the genus *Streptomyces* is one of the most prolific sources of chemically diverse small molecules [1]. Terrestrial surface soil is the well-known habitat of this genus, but, since *Streptomyces* have been extensively searched for several decades, discovery of strains producing novel compounds becomes difficult from easily accessible soil samples. Therefore, untapped sources such as plants have recently attracted attention to obtain new strains for new secondary metabolites [2, 3]. In our continuing search for structurally rare metabolites from *Streptomyces*, alchivemycins A and B, which have potent antimicrobial activity and inhibitory effects on tumor cell invasion, were discovered from a plant-derived *Streptomyces* strain TP-A0867. These compounds are novel polycyclic polyketides with an unprecedented carbon backbone [4, 5], however the biosynthetic gene cluster has not been known to date. In this study, we performed whole genome shotgun sequencing of the strain TP-A0867 to elucidate the biosynthetic pathway of alchivemycins. We herein present the draft genome sequence of *Streptomyces* sp. TP-A0867, together with the taxonomical identification of the strain, description of its genome properties, and annotation for secondary metabolite genes. The putative alchivemycin biosynthetic gene cluster and the plausible biosynthetic pathway are also described.

## Organism information

### Classification and features

**Fig. 1:**
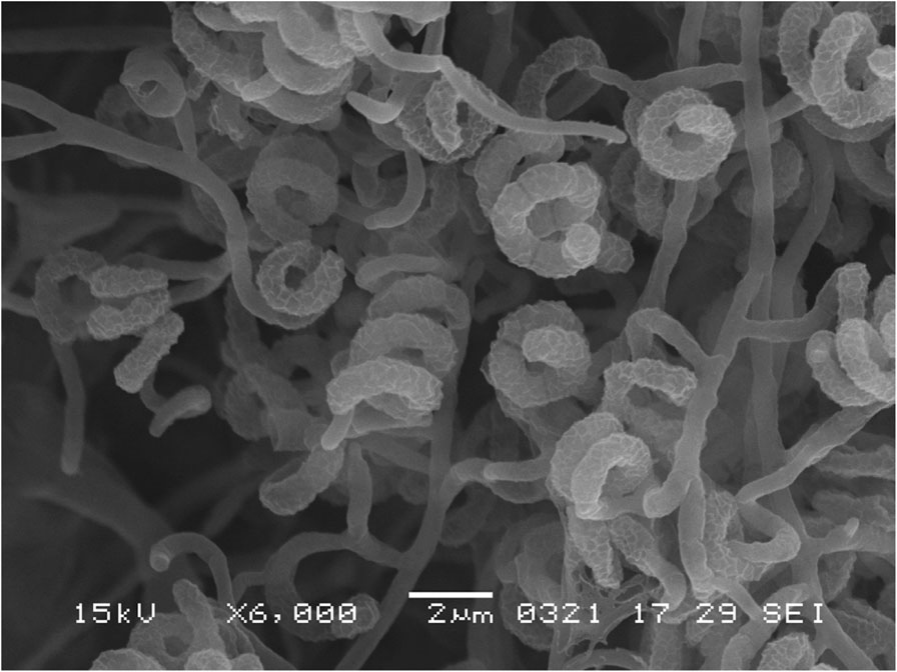
Scanning electron micrograph of *Streptomyces* sp. TP-A 0867 grown on double-diluted ISP 2 agar for 7 days at 28 °C. Bar, 2 μm

**Fig. 2:**
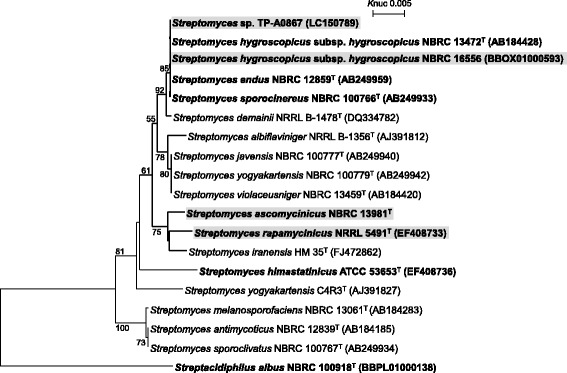
Phylogenetic tree of *Streptomyces* strains based on 16S rRNA gene sequences. The 16S rRNA sequences were obtained from GenBank, whose accession numbers are shown in parentheses, whereas that of *Streptomyces ascomycinicus* NBRC 13981^T^ was downloaded from ‘Sequence Information’ of the NBRC Culture Catalog Search (www.nbrc.nite.go.jp/NBRC2/SequencSearchServlet?ID=NBRC&CAT=00013981&DNA=2). The tree was constructed by the neighbor-joining method [45] using sequences aligned by ClustalX2 [9]. All positions containing gaps were eliminated. The building of the tree also involves a bootstrapping process repeated 1,000 times to generate a majority consensus tree, and only bootstrap values above 50 % are shown at branching points. *Streptacidiphilus albus* NBRC 100918^T^ was used as an outgroup. Strains whose genome were sequenced are boldfaced. Among the genome-sequenced strains, those harboring the putative alchivemycin biosynthetic gene cluster are shadowed in gray

In the course of screening for new bioactive compounds produced by plant-derived actinomycetes, *Streptomyces* sp. TP-A0867 was isolated from a leaf of a Chinese chive (*Allium tuberosum*) collected in Toyama, Japan [2] and two new polyketides, alchivemycins A and B, were found from its culture broth [4, 5]. The characteristics of *Streptomyces* sp. TP-A0867 were examined by the same manner of our previous report [6]. This strain grew well on ISP 2, ISP 4, and ISP 6 agar media, but poorly on ISP 5 and ISP 7. Colors of aerial mycelia were determined using the Japanese Industrial Standard Common Color Names (JIS Z 8102: 2001). The color of aerial mycelia was light gray and that of the reverse side was pale yellow on ISP 2 agar medium. No diffusible pigment was observed on ISP 2, ISP 3, ISP 4, ISP 5, ISP 6, and ISP 7 agar media. A scanning electron micrograph of this strain (Fig. 1) shows that spore chains were spiral and contained 2–3 helixes and 5–8 spores per chain; spores were cylindrical and 0.9 × 1.8 μm in size, and had a rugose ornamentation. Motile cells were not observed in hanging drops under a light microscope. Growth occurred at 15–45 °C (optimum 40 °C) on ISP 2 agar medium. Strain TP-A0867 exhibited growth with 0–5 % (w/v) NaCl (optimum 0 % NaCl) at 28 °C on ISP 2 agar medium and pH 4–10 (optimum pH 7) at 28 °C in ISP 2 liquid medium. Carbohydrate utilization was determined on Pridham-Gottlieb carbon utilization (ISP 9) agar medium supplemented with 1 % (w/v) of carbon sources sterilized by filtration. Strain TP-A0867 utilized fructose, glucose, rhamnose, sucrose, and xylose for growth. These results are summarized in Table 1. The genes encoding 16S rRNA were amplified by PCR using two universal primers, 9 F (5′-GAGTTTGATCCTGGCTCAG-3′) and 1541R (5′-AAGGAGGTGATCCAGCC-3′) [7]. KOD FX (Toyobo Co., Ltd., Tokyo, Japan) was used as described by the manufacturer for the PCR. The reaction was started with denaturation at 94 °C for 1 min followed by a total 30 cycles that consisted of denaturation at 98 °C for 10 s, annealing at 55.5 °C for 30 s, and extension at 68 °C for 1.5 min. The amplicon size was 1.5 kb. After purification of the PCR product by AMPure (Beckman Coulter), sequencing was carried out according to an established method [7]. The sequence was deposited into DDBJ under the accession number LC150789. BLAST search of the sequence by EzTaxon-e [8] indicated the highest similarities to those of *Streptomyces hygroscopicus* subsp. *hygroscopicus* NRRL 2387 ^T^ (AB231803, 100 %, 1456/1456), *Streptomyces endus* NRRL 2339 ^T^ (AY999911, 100 %, 1456/1456), and *Streptomyces sporocinereus* NBRC 100766 ^T^ (AB249933, 100 %, 1456/1456). A phylogenetic tree was reconstructed on the basis of the 16S rRNA gene sequence together with *Streptomyces* type strains showing over 98.5 % similarities and *S. hygroscopicus* subsp. *hygroscopicus* NBRC 16556 using ClustalX2 [9] and NJPlot [10] as shown in Fig. 2. The phylogenetic analysis confirmed that the strain TP-A0867 belongs to the genus *Streptomyces*.

**Table 1:** Classification and general features of *Streptomyces* sp. TP-A0867 [12]

MIGS ID	Property	Term	Evidence code^a^
	Classification	Domain *Bacteria*	TAS [34]
		Phylum *Actinobacteria*	TAS [35]
		Class *Actinobacteria*	TAS [36]
		Order *Actinomycetales*	TAS [36–39]
		Suborder *Streptomycineae*	TAS [36, 39]
		Family *Streptomycetaceae*	TAS [36, 38–41]
		Genus *Streptomyces*	TAS [38, 41–43]
		Species *Streptomyces hygroscopicus*	IDA
		Subspecies *Streptomyces hygroscopicus subsp. hygroscopicus*	IDA
		Strain TP-A0867	[4]
	Gram stain	Not tested, likely positive	NAS
	Cell shape	Branched mycelia	IDA
	Motility	Not observed	IDA
	Sporulation	Sporulating	IDA
	Temperature range	Grows from 15 °C to 45 °C	IDA
	Optimum temperature	40 °C	IDA
	pH range; Optimum	4 to 10; 7	IDA
	Carbon source	Fructose, glucose, rhamnose, sucrose, xylose	IDA
MIGS-6	Habitat	Chinese chive (*Allium tuberosum*)	TAS [2, 4]
MIGS-6.3	Salinity	Grows from 0 % to 7 % NaCl	IDA
MIGS-22	Oxygen requirement	Aerobic	IDA
MIGS-15	Biotic relationship	Free-living	IDA
MIGS-14	Pathogenicity	Not reported	
MIGS-4	Geographic location	Toyama, Japan	TAS [2]
MIGS-5	Sample collection	from April to June in 1998	TAS [2]
MIGS-4.1	Latitude	Not reported	
MIGS-4.2	Longitude	Not reported	
MIGS-4.4	Altitude	Not reported	

^a^Evidence codes - *IDA* Inferred from Direct Assay, *TAS* Traceable Author Statement (i.e., a direct report exists in the literature), *NAS* Non-traceable Author Statement (i.e., not directly observed for the living, isolated sample, but based on a generally accepted property for the species, or anecdotal evidence). These evidence codes are from the Gene Ontology project [44]

#### Chemotaxonomic data

Biomass for chemotaxonomic studies was obtained by cultivating strain TP-A0867 in shake flasks of ISP 2 broth for 2 days at 28 °C at 100 r.p.m. The isomer of diaminopimelic acid in the whole-cell hydrolysate was analyzed according to the method described by Hasegawa et al. [11]. Isoprenoid quinones and cellular fatty acids were analyzed as described previously [7]. The whole-cell hydrolysate of strain TP-A0867 contained ll-diaminopimelic acid as its diagnostic peptidoglycan diamino acid. The predominant menaquinones were identified as MK-9(H_2_) (33 %), MK-9(H_4_) (40 %) and MK-9(H_6_) (23 %). The major cellular fatty acids were found to be C_16:0_ (27 %), anteiso-C_15:0_ (18 %) and iso-C_15:0_ (12 %).

## Genome sequencing information

### Genome project history

In collaboration between Toyama Prefectural University and NBRC, the organism was selected for genome sequencing to elucidate the alchivemycin biosynthetic pathway. We successfully accomplished the genome project of *Streptomyces* sp. TP-A0867 as reported in this paper. The draft genome sequences have been deposited in the INSDC database under the accession numbers BBON01000001 to BBON01000259. The project information and its association with MIGS version 2.0 compliance are summarized in Table 2 [12].

**Table 2:** Project information

MIGS ID	Property	Term
MIGS 31	Finishing quality	Improved-high-quality draft
MIGS-28	Libraries used	Illumina paired-end library
MIGS 29	Sequencing platforms	Illumina MiSeq
MIGS 31.2	Fold coverage	98 x
MIGS 30	Assemblers	Newbler v2.6, GenoFinisher, Sequencher v5.1
MIGS 32	Gene calling method	Prodigal
	Locus tag	TPA0867
	GenBank ID	BBON00000000
	GenBank date of release	March 24, 2016
	GOLD ID	Not registered
	BioProject	PRJDB3206
MIGS 13	Source material identifier	NBRC 109436
	Project relevance	Industrial

### Growth conditions and genomic DNA preparation

*Streptomyces* sp. TP-A0867 was deposited in the NBRC culture collection with the registration number of NBRC 109436. Its monoisolate was grown on polycarbonate membrane filter (Advantec) on double diluted NBRC 227 agar medium (0.2 % yeast extract, 0.5 % malt extract, 0.2 % glucose, 2 % agar, pH 7.3) at 28 °C. High quality genomic DNA for sequencing was extracted and isolated from the mycelia with an EZ1 DNA Tissue Kit and a Bio Robot EZ1 (Qiagen) according to the manufacturer’s protocol for extraction of nucleic acid from Gram-positive bacteria. The size, purity, and double-strand DNA concentration of the genomic DNA were measured by agarose gel electrophoresis, ratio of absorbance values at 260 nm and 280 nm, and Quant-iT PicoGreen dsDNA Assay Kit (Life Technologies) to assess the quality. Two hundreds fifty ng of the genomic DNA were used for the preparations of Illumina paired-end library.

### Genome sequencing and assembly

A paired-end library with 500 bp insert was constructed and 130 bp from each end was sequenced using MiSeq (Illumina K.K., Tokyo, Japan) according to manufacturer’s protocols (Table 2). The 799 Mb paired-end sequences were assembled into 259 scaffolds larger than 500 bp using Newbler v2.6 (Roche Applied Science, Branford, CT, USA) with the default parameters. Subsequently, each sequence gap in scaffolds was checked and re-assembled using sequence reads belonging to gap extremes by GenoFinisher [13]. Branching contigs, one connected to multiple other contigs, were also examined and misassembled linkages were corrected. The sequences of the alchivemycin biosynthetic gene cluster were further checked manually by Sequencher v.5.1 (Gene Codes Corporation, Ann Arbor, MI, USA)

### Genome annotation

Coding sequences were predicted with Prodigal [14] and tRNA-scanSE [15]. The gene functions were assigned using an in-house genome annotation pipeline, and searched for domains related to polyketide synthase (PKS) and nonribosomal peptide synthetase (NRPS) using the SMART and PFAM domain databases [16, 17]. PKS and NRPS gene clusters and their domain organizations were determined as reported previously [18]. Similarity search results against the NCBI non-redundant database were also used for predicting function of genes in the biosynthetic gene clusters.

## Genome properties

The total size of the genome is 9,889,163 bp and the GC content is 71.9 % (Table 3), similar to other genome-sequenced *Streptomyces* members. Of the total 8,453 genes, 8,385 are protein-coding genes and 68 are RNA genes. The classification of genes into COGs functional categories is shown in Table 4. As for the synthesis of secondary metabolites such as polyketides and nonribosomal peptides, this genome encodes at least five type I PKS gene clusters, one type II PKS gene cluster, four NRPS gene clusters, and two hybrid PKS/NRPS gene clusters. This suggests the potential to produce diverse polyketide- and nonribosomal peptide-compounds as the secondary metabolites. Two type I PKS gene clusters are putatively identified for syntheses of nigericin and geldanamycin, respectively, and one hybrid PKS/NRPS gene cluster could be responsible for alchivemycin synthesis as stated below. The others are orphan gene clusters at present.

**Table 3:** Genome statistics

Attribute	Value	% of Total
Genome size (bp)	9,889,163	100.0
DNA coding (bp)	8,515,958	85.2
DNA G + C (bp)	7,107,274	71.8
DNA scaffolds	259	-
Total genes	8,453	100.0
Protein coding genes	8,385	99.2
RNA genes	68	0.8
Pseudogenes	-	-
Genes in internal clusters	3,697	44.1
Genes with function prediction	5,588	66.1
Genes assigned to COGs	7,232	86.2
Genes with Pfam domains	6,077	71.9
Genes with signal peptides	625	7.4
Genes with transmembrane helices	1,629	19.3
CRISPR repeats	3	-

**Table 4:** Number of genes associated with general COG functional categories

Code	Value	% age	Description
J	289	3.4	Translation, ribosomal structure and biogenesis
A	4	0.04	RNA processing and modification
K	1,036	12.3	Transcription
L	345	4.1	Replication, recombination and repair
B	3	0.03	Chromatin structure and dynamics
D	52	0.6	Cell cycle control, Cell division, chromosome partitioning
V	133	1.58	Defense mechanisms
T	506	6.03	Signal transduction mechanisms
M	337	4.02	Cell wall/membrane biogenesis
N	43	0.51	Cell motility
U	85	1.01	Intracellular trafficking and secretion
O	211	2.52	Posttranslational modification, protein turnover, chaperones
C	543	6.48	Energy production and conversion
G	751	8.96	Carbohydrate transport and metabolism
E	811	9.67	Amino acid transport and metabolism
F	134	1.60	Nucleotide transport and metabolism
H	286	3.41	Coenzyme transport and metabolism
I	431	5.14	Lipid transport and metabolism
P	462	5.51	Inorganic ion transport and metabolism
Q	529	6.31	Secondary metabolites biosynthesis, transport and catabolism
R	1,383	16.5	General function prediction only
S	471	5.62	Function unknown
-	1,153	13.8	Not in COGs

The total is based on the total number of protein coding genes in the genome

## Insights from the genome sequence

### Taxonomic identification of *Streptomyces* sp. TP-A0867

The 16S rRNA gene sequence of *Streptomyces* sp. TP-A0867 was identical to those of *S. hygroscopicus* subsp. *hygroscopicus* NBRC 13472 ^T^ (AB184428), *S. hygroscopicus* subsp. *hygroscopicus* NBRC 16556 (BBOX01000593), *S. endus* NBRC 12859 ^T^ (AB249959), and *S. sporocinereus* NBRC 100766 ^T^ (AB249933). To determine the scientific name of the strain TP-A0867, we calculated average nucleotide identity based on BLAST values between strain TP-A0867 and the three type strains using their genome sequences (NBRC 13472, BBOX00000000; NBRC 12859, BBOY00000000; NBRC 100766, BCAN00000000) using JSpecies [19]. The ANIb values between *Streptomyces* sp. TP-A0867 and the type strains of *S. hygroscopicus* subsp. *hygroscopicus*, *S. endus*, and *S. sporocinereus* were 97.16 %, 97.10 %, and 98.54 %, respectively. Since these values are above the threshold (95–96 %) corresponding to DNA relatedness value of 70 % recommended as the cut-off point for the assignment of bacterial strains to the same species [19, 20], strain TP-A0867 can be classified into these three taxa. We also analyzed the *in silico* DNA-DNA hybridization values using these genome sequences with a different and quickly method provided from the DSMZ website [21]. The analysis estimated that the DDH values between *Streptomyces* sp. TP-A0867 and the three type strains were 76.2 %, 76.2 %, and 87.6 %, respectively, supporting our results clearly. Once this strain was reported to be *S. endus* [22], however *S. endus* and *S. sporocinereus* were reported as the later heterotypic synonyms of *S. hygroscopicus* subsp. *hygroscopicus* in 2012 [23], although the taxonomic proposal has not been validated. Therefore, we classified strain TP-A0867 into *S. hygroscopicus* subsp. *hygroscopicus* as shown in Table 1.

### Proposal of alchivemycin biosynthetic pathway

**Fig. 3:**
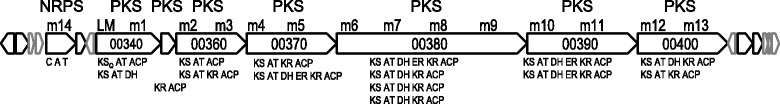
Genetic map of the putative alchivemycin biosynthetic gene cluster (TPA0867_155_00270 to TPA0867_155_00460) of *Streptomyces* sp. TP-A0867

**Fig. 4:**
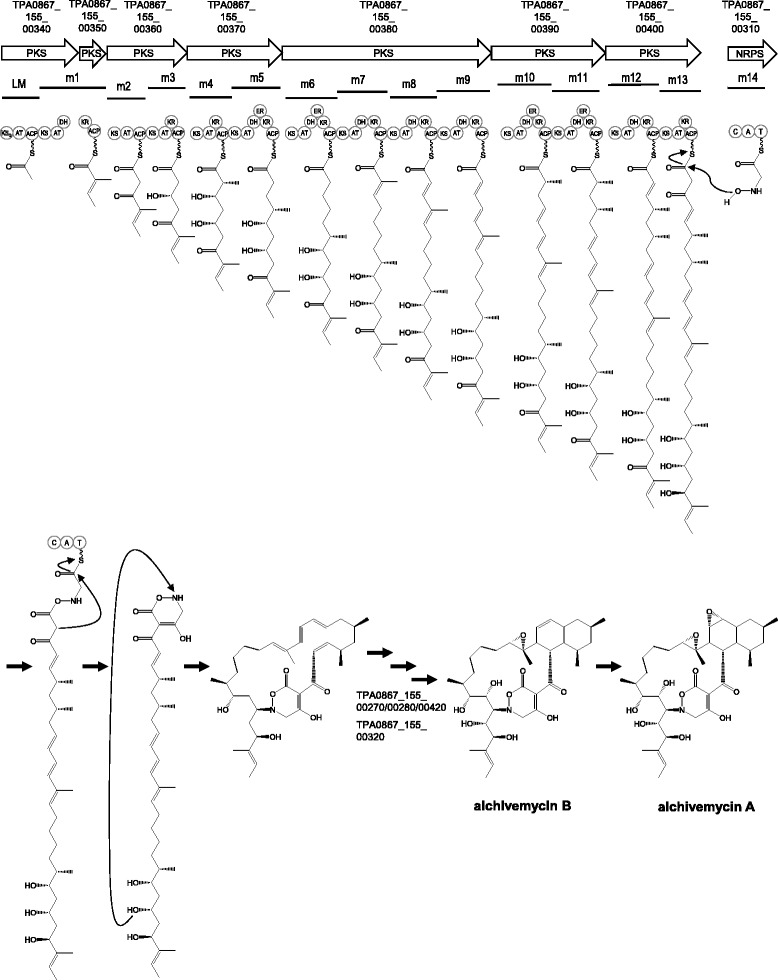
Proposed alchivemycin biosynthetic pathway

Our previous study suggested that the carbon backbone of alchivemycins is assembled from five methylmalonyl-CoA, nine malonyl-CoA and one glycine molecules by a hybrid PKS/NRPS pathway [5]. We therefore searched for a hybrid PKS/NRPS gene cluster consisting of fourteen PKS modules and one NRPS module and, indeed, a hybrid PKS/NRPS gene cluster was found in scaffold00155 (Table 5, Fig. 3) that consisted of fourteen PKS modules and one NRPS module (Fig. 4), while no other such gene clusters are present in the genome. Almost all domains in each module conserved active residues and/or signature sequences defined in the previous report [24], but the first ketosynthase (KS) domain in TPA0867_155_00340 had glutamine substituted for the active site cysteine residue, suggesting this domain is KSQ [25, 26] and this module is for loading starter molecule in this assembly line. The acyltransferase (AT) domains of modules 1, 4, 7, 10, and 11 were predicted to load a methylmalonyl-CoA in the elongating polyketide chain, because they have YASHS as signature amino-acid residues specific for methylmalonyl-CoA [27, 28]. In contrast, the remaining nine AT domains were predicted to load a malonyl-CoA since the diagnostic residues HAFHS, specific for malonyl-CoA, were found; although that of module 2 is not HAFHS but RAFHS. These results suggest that the PKS assembly line synthesizes a polyketide chain by sequential incorporation of C_2_-C_3_-C_2_-C_2_-C_3_-C_2_-C_2_-C_3_-C_2_-C_2_-C_3_-C_3_-C_2_-C_2_ units, consistent with our previous ^13^C-labeled precursor feeding experiments [5]. In the PKS assembly line, combination of optional domains such as ketoreductase (KR), dehydratase (DH) and enoylreductase (ER) between AT and acyl carrier protein in each module determines reduction of the ketone group, dehydration of the resulting hydroxyl group and subsequent reduction of the double bond, respectively [29]. PKS modules in the PKS/NRPS gene cluster have three KRs, five DH/KR pairs and four DH/ER/KR trios, corresponding to hydroxyl group, double bond, and saturated carbon, respectively, as the optional domains. We also analyzed signature sequences of KR and ER domains to predict absolute configuration of secondary hydroxyl groups derived from acyl keto groups and methyl branches derived from methylmalonyl-CoA based on the fingerprinting and flowchart reported previously [30, 31]. Based on these experimental and bioinfomatic analyses, a putative linear polyketide precursor of alchivemycin for macrocyclization is shown under module 13 (m13) in Fig. 4, which is in good accordance with the carbon backbone of alchivemycins. Alchivemycin contains an unusual heterocyclic system tetrahydrooxazine ring that derives from glycine-incorporation [5]. A gene encoding NRPS (TPA0867_155_00310) is present upstream the PKS genes (Fig. 3), and the substrate of its adenylation (A) domain was predicted to be glycine by the PKS/NRPS Analysis Web-site (http://nrps.igs.umaryland.edu/nrps/) [32]. This strongly supports the idea that this NRPS is involved in the glycine uptake into the tetrahydrooxazine ring: Kim et al. found that the ^13^C-labeled glycine was actually incorporated into the heterocyclic part of alchivemycin A [5]. After the tetrahydrooxazine ring formation, modifications such as cyclization, epoxidation, and oxidation may take place as shown in Fig. 4. Three monooxygenases (TPA0867_155_00270, TPA0867_155_00280 and TPA0867_155_00420) and a cytochrome P450 (TPA0867_155_00320) are encoded in this cluster, but it was unable to determine which enzymes catalyze the epoxidation at two positions and oxidation at C-24 only by bioinformatic analyses. On the basis of the above-mentioned bioinfomatic evidences, we propose that this PKS/NRPS gene cluster could be responsible for the synthesis of alchivemycins. Further experiments including gene-disruption to prove this proposal are currently in progress.

**Table 5:** ORFs in the putative alchivemycin-biosynthetic gene cluster of *Streptomyces* sp. TP-A0867

TPA0867_155_ (locus tag)	Length (aa)	Deduced function	Protein homolog [origin]	Identity/similarity (%)	Accession number
**00270** ^**a**^	**526**	**monooxygenase**	hypothetical protein M271_21675 [*S. rapamycinicus* NRRL 5491]	96/97	AGP55859
**00280**	**510**	**monooxygenase**	hypothetical protein M271_21670 [*S. rapamycinicus* NRRL 5491]	95/96	AGP55858
00290	197	unknown	hypothetical protein M271_21665 [*S. rapamycinicus* NRRL 5491]	97/99	AGP55857
00300	270	unknown	hypothetical protein M271_21660 [*S. rapamycinicus* NRRL 5491]	95/96	AGP55856
**00310**	**1,117**	**NRPS**	hypothetical protein M271_21655 [*S. rapamycinicus* NRRL 5491]	88/89	AGP55855
**00320**	**405**	**cytochrome P450**	hypothetical protein M271_21650 [*S. rapamycinicus* NRRL 5491]	96/97	AGP55854
00330^a^	293	oxidoreductase	hypothetical protein M271_21645 [*S. rapamycinicus* NRRL 5491]	98/99	AGP55853
**00340**	**2,516**	**PKS**	hypothetical protein M271_21640 [*S. rapamycinicus* NRRL 5491]	87/90	AGP55852
**00350**	**578**	**PKS**	hypothetical protein M271_21640 [*S. rapamycinicus* NRRL 5491]	90/92	AGP55852
**00360**	**2,890**	**PKS**	type I polyketide synthase AVES 4 [*Streptomyces avermitilis* MA-4680]	54/63	NP_822118
**00370**	**3,731**	**PKS**	hypothetical protein M271_21625, partial [*S. rapamycinicus* NRRL 5491]	89/91	AGP55849
**00380**	**7,654**	**PKS**	AmphC [*Streptomyces nodosus*]	53/64	AAK73514
**00390**	**4,354**	**PKS**	hypothetical protein M271_21600, partial [*S. rapamycinicus* NRRL 5491]	87/90	AGP55844
**00400**	**3,637**	**PKS**	beta-ketoacyl synthase [*S. violaceusniger* Tu 4113]	54/64	YP_004817601
00410^a^	309	phytanoyl-CoA dioxygenase	hypothetical protein M271_21580 [*S. rapamycinicus* NRRL 5491]	93/96	AGP55840
**00420**	**608**	**monooxygenase**	hypothetical protein M271_21575 [*S. rapamycinicus* NRRL 5491]	91/93	AGP55839
00430	426	transcriptional regulator	helix-turn-helix domain-containing protein [*S. violaceusniger* Tu 4113]	93/95	YP_004817135
00440	199	unknown	hypothetical protein [*S. violaceusniger* Tu 4113]	75/80	YP_004812903
00450	157	unknown	hypothetical protein M271_33560 [*S. rapamycinicus* NRRL 5491]	56/64	AGP58126
00460	295	phosphotransferase	aminoglycoside phosphotransferase [*S. violaceusniger* Tu 4113]	71/78	YP_004817724

^a^encoded in complementary strand. Genes shown in Fig. 4 are bold-faced

### Distribution of putative alchivemycin biosynthetic gene clusters in other strains

**Fig. 5:**
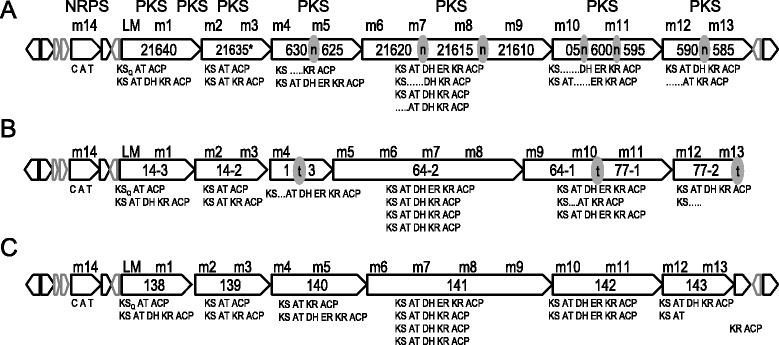
Genetic maps of putative alchivemycin biosynthetic gene clusters of *S. rapamycinicus* NRRL 5491 (**a** M271_21675 to M271_21575), *S. hygroscopicus subsp. hygroscopicus* NBRC 16556 (**b** orf10 to orf1 in scaffold14, orf3 to orf1 in scaffold64, and orf1 to orf2 in scaffold77), and *S. ascomycinicus* NBRC 13981^T^ (**c** orf131 to orf145 of scaffold16). n (in grey circle), these parts contained many undetermined DNA sequences; t (in grey circle), scaffold terminal because **b** was not obtained as single scaffold. *We manually annotated the ORF, which were longer than registered in GenBank/EMBL/DDBJ

BLAST search of ORFs in the putative alchivemycin gene cluster within the NCBI database suggested that a similar gene cluster is present in *Streptomyces rapamycinicus* NRRL 5491 ^T^ because this strain has several protein homologues with high sequence homology (Table 5). Analysis of secondary metabolite gene clusters in the genome of strain NRRL 5491 ^T^ revealed that a gene cluster from M271_21585 to M271_21655 and the PKS/NRPS domain organizations are identical between *Streptomyces* sp. TP-A0867 (Fig. 3) and *S. rapamycinicus* NRRL 5491 ^T^ (Fig. 5a), although the genome sequence of the strain NRRL 5491 ^T^ is incomplete and its cluster sequence contains several undetermined DNA sequence regions. This finding prompted us to investigate distribution of putative alchivemycin biosynthetic gene clusters in other *Streptomyces* strains. Further BLAST search of putative alchivemycin-biosynthetic genes indicated that the gene cluster is also present in *S. hygroscopicus* subsp. *hygroscopicus* NBRC 16556 (Fig. 5b) and *Streptomyces ascomycinicus* NBRC 13981 ^T^ (Fig. 5c). These strains are phylogenetically close to strain TP-A0867 (Fig. 2, shaded in gray), suggesting that putative alchivemycin-biosynthetic pathway is likely specific in this taxonomical group highlighted by bold lines, although it is unclear whether strains whose genome sequences are unavailable, not boldfaced in the phylogenetic tree, harbor the pathway at present. All the four clusters of *Streptomyces* sp. TP-A0867, *S. rapamycinicus* NRRL 5491 ^T^, *S. hygroscopicus* subsp. *hygroscopicus* NBRC 16556, and *Streptomyces ascomycinicus* NBRC 13981 ^T^ show conserved synteny, and encode all essential enzymes such as PKSs, an NRPS, and P450/monooxygenases likely for alchivemycin synthesis (Figs. 3 and 5). These results suggest that these strains might also have potential to produce alchivemycins.

### Alchivemycin production by *S. ascomycinicus*NBRC 13981^T^

We examined alchivemycin production of *S. hygroscopicus* subsp. *hygroscopicus* NBRC 16556 and *S. ascomycinicus* NBRC 13981 ^T^, both of which are available from the NBRC culture collection. However, the production was not reproducibly observed in some liquid culture conditions tested in this study. Then, we attempted to obtain mutants that can stably produce alchivemycins. *S. ascomycinicus* NBRC 13981 ^T^ was inoculated and cultured on potato dextrose agar (PDA) medium (Merck & Co.) to obtain single colonies, and then the subculture was continuously performed using PDA medium. Within five generations of the subculture, bald mutants were observed. The bald mutants were isolated and maintained on PDA medium to check bald phenotype. Each mutant was cultured using PDA medium for 7 days at 30 °C. The mycelial cells were harvested by steel spatula, and the cells were extracted by equal volume of methanol (MeOH). After centrifugation to remove insoluble materials, the MeOH extracts were analyzed by HPLC coupled with ESI-MS to detect alchivemycins. The alchivemycin production was observed in the MeOH extract of a mutant strain designated as T3. Since loss of morphological differentiation leads to loss of secondary metabolite production in *Streptomyces* [33], it is generally recognized that bald mutants lose their ability to produce secondary metabolites. Our result differs from such an empirical recognition. We also deposited the bald mutant to the NBRC culture collection and the comparative genome analysis is in progress.

## Conclusions

The 9.9 Mb draft genome of *Streptomyces* sp. TP-A0867, a producer of alchivemycins isolated from a leaf of a Chinese chive, has been deposited at GenBank/ENA/DDBJ under the accession number BBON00000000. This strain was identified to be *S. hygroscopicus* subsp. *hygroscopicus*. We successfully identified a putative PKS/NRPS hybrid gene cluster that could be for alchivemycin synthesis and proposed the plausible biosynthetic pathway. Alchivemycin biosynthetic gene clusters are also present in the genomes of taxonomically close strains, one of which was able to produce alchivemycins. The genome sequence information disclosed in this study will be utilized for the investigation of additional new bioactive compounds and will also serve as a valuable reference for evaluation of the metabolic potential in plant-derived *Streptomyces*.

## Abbreviations

A: Adenylation
ACP: Acyl carrier protein
ANI: Average nucleotide identity
ANIb: ANI based on BLAST
AT: Acyltransferase
BLAST: Basic Local Alignment Search Tool
C: Condensation
CoA: Coenzyme A
DDBJ: DNA Data Bank of Japan
DDH: DNA-DNA hybridization
DH: Dehydratase
ER: Enoylreductase
ESI: Electrospray ionization
HPLC: High-performance liquid chromatography
INSDC: International Nucleotide Sequence Database Collaboration
ISP: International *Streptomyces* Project
KR: Ketoreductase
KS: Ketosynthase
KSQ: KS-like domain with glutamine substituted for the active site cysteine residue
LM: Loading module
m: Module
MeOH: Methanol
MIGS: The minimum information about a genome sequence
MS: Mass spectrometer
NBRC: Biological Resource Center, National Institute of Technology and Evaluation
NCBI: National Center for Biotechnology Information
NRPS: Nonribosomal peptide synthetase
PDA: Potato dextrose agar
PKS: Polyketide synthase
T: Thiolation
